# Interferon Therapy in Acute Myeloid Leukemia: Biological Rationale, Clinical Evidence, and Future Directions

**DOI:** 10.3390/cancers18203266

**Published:** 2026-10-10

**Authors:** Jan Philipp Bewersdorf, Hsin-An Hou, Evelyn Yingtzu Yen, Shinya Imada, Shaoyu Chang, Xavier Cheng-Hong Tsai, Albert Qin

**Affiliations:** 1 Section of Medical Oncology and Hematology, Department of Internal Medicine, Yale School of Medicine and Yale Comprehensive Cancer Center, New Haven, CT 06510, USA; 2 Division of Hematology, Department of Internal Medicine, National Taiwan University Hospital, Taipei 100215, Taiwanxavierchtsai@ntu.edu.tw (X.C.-H.T.); 3 PharmaEssentia Corporation, Taipei 115603, Taiwan; 4 PharmaEssentia USA, Burlington, MA 01803, USA

**Keywords:** Interferon therapy, acute myeloid leukemia, immunotherapy, leukemic stem cell, measurable residual disease

## Abstract

Acute myeloid leukemia (AML) is a heterogeneous hematologic malignancy characterized by uncontrolled clonal proliferation of immature myeloid blasts in bone marrow and peripheral blood. The contemporary therapeutic landscape for AML spans a broad spectrum of interventions, relying on cytotoxic chemotherapy, hypomethylating agents, and an expanding array of targeted therapies to accommodate both patients eligible for intensive induction and those requiring lower intensity regimens. Allogeneic hematopoietic stem cell transplantation is performed with curative intent in eligible high-risk patients, and donor lymphocyte infusion is utilized to prevent or treat post-transplant early relapse. Despite ongoing therapeutic advances, the overall prognosis for AML remains unsatisfactory with a five-year relative survival rate of approximately 31.9%. Driven by an evolving understanding of AML biology, particularly the roles of leukemic stem cells and measurable residual disease, interferon (IFN) therapy, specifically type I and type II IFNs, has emerged as a compelling therapeutic candidate, distinguished by its direct anti-leukemic effect and indirect anticancer activities, notably via immunomodulatory effects. This review outlines the biological rationale and clinical evidence for current IFN therapy in AML, aiming to inform future preclinical and clinical investigations.

## Simple Summary

Interferons (IFNs) are a class of signaling proteins that regulate gene expression critical for cell cycle progression and immune system function. Although IFN therapy has an established history in treating other myeloid neoplasms, its role in acute myeloid leukemia (AML) remains investigational. AML represents an aggressive blood cancer driven by diverse genetic mutations and characterized by bone marrow failure. Current AML management includes chemotherapy to eliminate leukemia cells and targeted therapies for patients harboring specific genetic profiles. Although stem cell transplantation offers a potential cure for eligible candidates, overall clinical outcomes remain suboptimal. Optimized IFN therapy could fill in the gap as a potential strategy for the direct killing of leukemic cells and resistant clones while inducing indirect immunomodulation, especially in delicate settings such as maintenance or pre-emptive therapy. This review synthesizes the biological rationale and clinical evidence for the use of IFN therapy, specifically type I and type II IFNs, in AML to inform future preclinical and clinical studies.

## 1. Introduction

Acute myeloid leukemia (AML) is an aggressive hematologic malignancy marked by excessive clonal proliferation and arrested differentiation of myeloid blasts, leading to their accumulation in the bone marrow and peripheral blood, disruption of normal hematopoiesis, and eventually fatal complications related to bone marrow failure and/or complications of proliferative disease [1,2]. AML accounts for 80% of acute leukemias in adults [3]. In 2021, global incidence was estimated at approximately 144,600 cases, with the highest national burdens observed in the United States (21,533 cases), China (17,835 cases), and India (11,040 cases) [4]. Yet prognosis remains poor, with over 80,000 deaths annually worldwide and a five-year relative survival of only 31.9% [1,5]. Over the past three decades, its global incidence and mortality have surged by over 70% [6]. In the United States alone, it is projected to represent 1% of all new cancer cases and more than 11,000 deaths in 2026 [7]. Together, these trends underscore the urgent need for a deeper understanding of disease biology and for effective therapeutic strategies.

The disease manifestations of AML arise from the sequential acquisition of genetic and epigenetic alterations in hematopoietic stem and progenitor cells, which is closely related to age but can also arise in the context of a germline predisposition as well as exposure to potentially leukemogenic agents [8,9]. At the molecular level, recurrent mutations in genes such as *FLT3*, *NPM1*, *DNMT3A*, and *IDH1/IDH2*, chromosomal translocations including *RUNX1-RUNX1T1*, *CBFB-MYH11*, *PML-RARA*, and epigenetic changes all contribute to the persistent proliferative advantage and impair hematopoietic differentiation, aggravating malignant transformation [1,10]. The cumulative burden of aberrations gives rise to leukemic stem cells (LSCs), a rare, self-renewing population whose relative quiescence renders them poorly responsive to conventional chemotherapy [11,12,13]. The surviving chemoresistant LSCs establish a subclinical reservoir quantified as measurable residual disease (MRD), which ultimately drives disease relapse [14,15,16,17]. Moreover, the changes in immune microenvironment, including epigenetic downregulation and concurrent upregulation of inhibitory checkpoint ligands allow these leukemic blasts and LSCs to escape immune detection [18,19]. In contemporary AML management, genetic heterogeneity, LSC biology, and MRD status are increasingly acknowledged for shaping treatment decisions [14,20,21,22].

While cytotoxic chemotherapy, hypomethylating agents (HMAs), and targeted therapies remain the pillars of AML treatment, long-term survival is commonly hindered by treatment resistance and residual disease that ultimately leads to disease relapse and recurrence. Although outcomes have gradually improved over time, five-year overall survival (OS) remains suboptimal ranging from 40–50% in patients under 60 years to 15–20% in those over 60, and falling to three to seven months once relapsed or refractory disease develops [23,24,25,26,27,28]. The median OS in older patients with age 65 or above has been reported to be as short as 2 months [29,30,31,32].

Allogeneic hematopoietic stem cell transplantation (allo-HSCT) is a potentially curative therapeutic option but only a subset of patients is eligible due to age, comorbidities and failure to achieve sufficient disease control. Even among allo-HSCT recipients disease relapse is common occurring in up to 30–40% of AML patients and being associated with a dismal two-year survival rate of less than 20% [33,34,35,36]. Hence, interventions to improve outcomes after allo-HSCT and reduce relapse rates are urgently needed. Currently available options include donor lymphocyte infusions (DLIs), though their comparative clinical benefit remains a matter of ongoing debate [37]. Furthermore, standard intensive chemotherapy is often poorly tolerated by special populations, particularly the elderly, who are most severely affected by the disease [32,38]. Overall, limitations persist across the entire spectrum of AML treatment, encompassing induction, preemptive, maintenance, and salvage therapies. These clinical challenges highlight an unmet need for mechanistically distinct and low-toxicity therapeutic strategies.

Interferons (IFNs) are a diverse family of pleiotropic cytokines broadly classified into three distinct categories based on their receptor specificities, encompassing type I (predominantly IFN-α, IFN-β), type II (IFN-γ), and type III (IFN-λ) [39,40]. Throughout the 1980s and 1990s, IFN-α was the mainstay of immunotherapy for hematological malignancies, most notably transforming the prognosis of hairy cell leukemia and serving as the standard of care for chronic myeloid leukemia (CML) [41,42,43,44]. However, its broad use in CML was largely replaced by tyrosine kinase inhibitors (TKIs) owing to their higher response rate and fewer side effects [45]. Building on this legacy, type I IFNs are currently under clinical evaluation in AML, while type II IFN is also gaining therapeutic interest, albeit at a more exploratory stage. Notably, no interferon is currently approved for the treatment of AML. Their direct anti-leukemic and immunomodulatory effects offer potential therapeutic promise, further supported by the manageable toxicity profiles associated with long-acting formulations [46,47,48,49,50,51,52,53,54]. Reflecting its extensive clinical track record in myeloid neoplasms, type I IFNs, particularly IFN-α, form the primary focus of this review, with IFN-γ considered briefly. Conversely, IFN-λ is omitted, as its lack of involvement in AML is likely attributable to its receptor expression being primarily restricted to epithelial cells [55].

This review provides an updated synthesis of the evolving clinical evidence for IFN therapy, specifically type I and type II IFNs, across the continuum of AML care. We elaborate on its biological rationale, current clinical studies, and future directions. Ultimately, we aim to contextualize the clinical potential of IFN therapy to inform treatment strategies.

## 2. The Biological Rationale for IFN Therapy in AML

**Figure 1:**
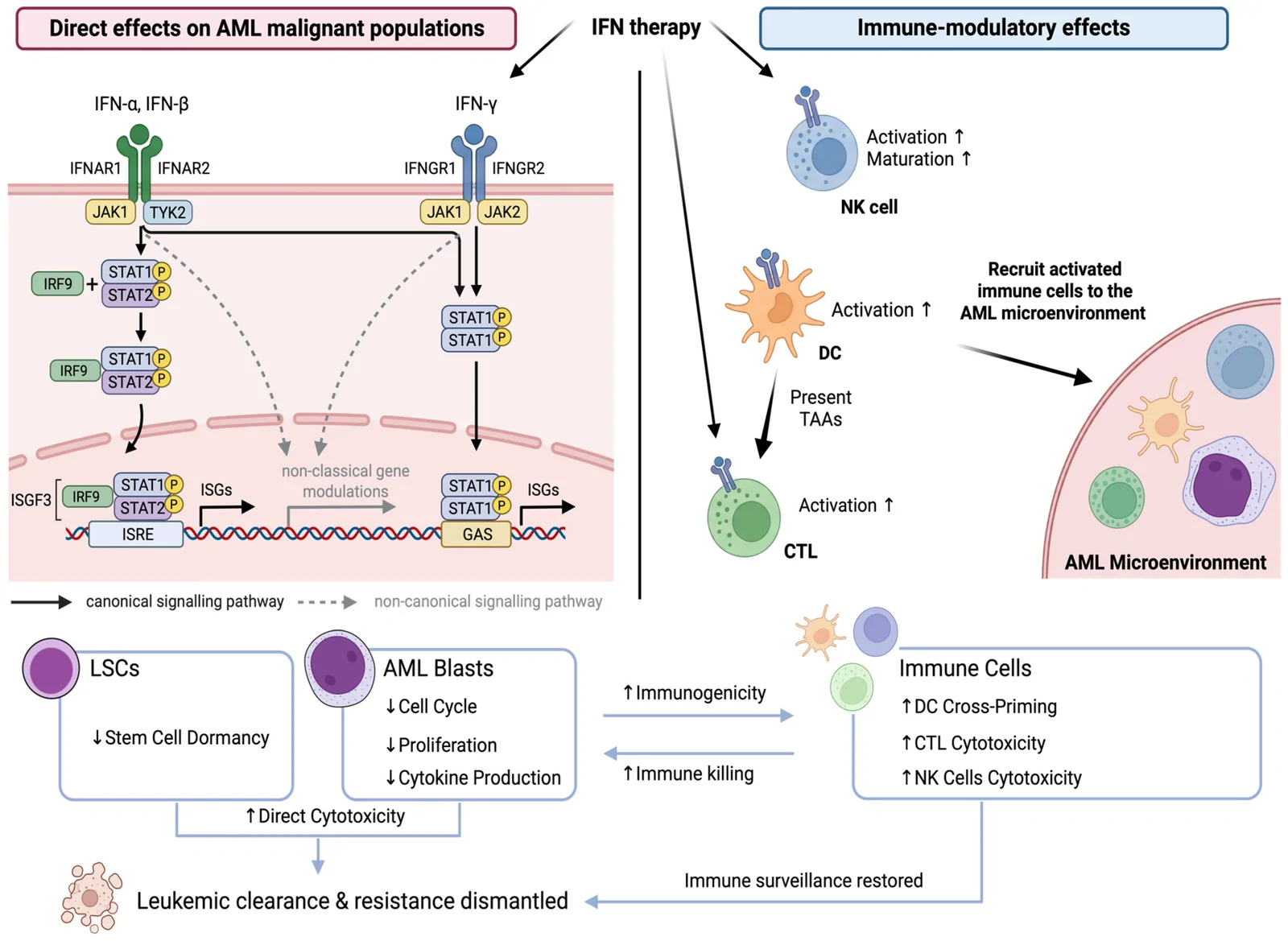
Mechanisms of interferon therapy (IFN) in acute myeloid leukemia (AML). IFN therapy exerts anti-leukemic activity through two complementary arms. (**Left**) Direct effects on AML malignant populations. Type I IFNs (IFN-α, IFN-β) bind the heterodimeric IFNAR1/IFNAR2 receptor, activating the receptor-associated kinases JAK1 and TYK2. Phosphorylated STAT1 and STAT2 associate with IRF9 to form the ISGF3 complex, which translocates to the nucleus and binds IFN-stimulated response elements (ISREs) to drive transcription of IFN-stimulated genes (ISGs). Type II IFN (IFN-γ) signals through IFNGR1/IFNGR2 and JAK1/JAK2, inducing STAT1 homodimers that bind gamma-activated sequences (GAS) to induce a distinct ISG program. Solid arrows denote canonical JAK–STAT signaling; dashed arrows denote non-canonical pathways that mediate non-classical gene modulation. (**Right**) Immune-modulatory effects on immune cells. IFN signaling enhances NK cell activation and maturation, promotes dendritic cell (DC) activation, and augments DC presentation of tumor-associated antigens (TAAs) to cytotoxic T lymphocytes (CTLs), thereby increasing CTL activation. Activated effector cells are recruited to the AML microenvironment. (**Bottom**) Integrated outcomes. Within the malignant compartment, IFNs reduce leukemic stem cell (LSC) dormancy, rendering quiescent LSCs susceptible to elimination, and suppress AML blast cell cycle progression, proliferation, and cytokine production, collectively increasing direct cytotoxicity. In parallel, IFN-mediated upregulation of blast immunogenicity enhances DC cross-priming and CTL and NK cell cytotoxicity, which in turn potentiates immune-mediated killing of leukemic cells. Together, preclinical evidence suggests that these mechanisms may restore immune surveillance, promote leukemic clearance, and dismantle therapeutic resistance. Created in BioRender. Yen, E. (2026) https://BioRender.com/9uvm2df.

The biological rationale for using IFNs in AML is hypothesized to stem from their pleiotropic properties, suggested to extend beyond simple cytotoxicity (Figure 1).

Canonically, type I IFNs signal through the IFNAR1/IFNAR2 to activate Janus kinase 1 (JAK1) and tyrosine kinase 2 (TYK2). These kinases phosphorylate signal transducer and activator of transcription (STAT) proteins STAT1 and STAT2, which complex with interferon regulatory factor 9 (IRF9) to form interferon stimulated gene factor 3 (ISGF3). ISGF3 then translocates to the nucleus, binding interferon-stimulated response elements (ISREs) in target promoters [56,57,58]. While it can share the capacity to induce promoter binding of the complete ISGF3 complex (STAT1, STAT2, and IRF9), IFN-γ signals via IFNGR1/IFNGR2 to activate JAK1 and JAK2, thereby triggering STAT1 homodimerization and binding to gamma-activated sequences (GAS) [39,59]. Both cascades, alongside non-canonical signaling pathways such as p38 mitogen-activated protein kinases (MAPK) and phosphatidylinositol 3-kinase (PI3K), culminate in the robust transcription of diverse ISGs [56,57,60]. Furthermore, by facilitating the efficient assembly of IFN-γ-activated transcription factors, IFNAR1 establishes a signaling crosstalk between the two classes of IFNs that provides a molecular basis for their overlapping biological functions [61]. By translating these molecular signals into broader phenotypic shifts within the tumor microenvironment, the resulting ISG network forms the core biological rationale for IFN therapy, operating through both direct anti-leukemic and immunomodulatory effects.

### 2.1. Direct Anti-Leukemic Effects

Direct action on AML blasts serves as the converging core mechanism for different types of IFNs [47,62,63]. Type 1 IFNs can either induce the cell cycle arrest at the G1 to S transition or inhibit the S-phase progression accompanied by a loss of tumorigenicity via the activation of an S-phase checkpoint in cancer cells [64,65]. Cell cycle alteration in specific types of cancer cells is determined by IFN signaling-induced gene regulations as well as the cell cycle regulatory components that are present in the cancer cells [65]. In hematopoietic cancer cells, type 1 IFN treatment typically causes growth arrest at the G1/S phase transition. Upon receptor engagement, IFN signaling rapidly downregulates the oncogenic transcription factor *c-Myc*. STAT-induced RIG-G expression suppresses c-Myc, leading to accumulation of the cyclin-dependent kinase (CDK) inhibitors p21 and p27. These inhibitors subsequently bind and inactivate the Cyclin E/CDK2 complex, causing cell cycle arrest at G1/S transition and hindering cell proliferation [66,67,68,69,70]. Type 1 IFNs induce malignant cell apoptosis and senescence [71,72]. Apoptosis is triggered by IFN therapy via death receptor ligation (e.g., Fas/CD95 or TRAIL), recruiting Fas-associated death domain (FADD) to activate caspase-8. This initiates mitochondrial cytochrome *c* release, loss of mitochondrial potential, plasma membrane asymmetry, and DNA fragmentation, followed by phagocytosis [73,74,75,76]. IFN-α also downregulates human telomerase reverse transcriptase (hTERT), abrogating the telomere compensation mechanism in malignant cells, restoring the mitotic clock function, and ultimately driving the cells toward replicative senescence or crisis [77].

Within the context of cell cycle regulation, IFN therapy has garnered increasing attention for its ability to target the protective LSC reservoir [14,16]. Similar to HSCs, LSCs reside within the hypoxic bone marrow niche maintaining a quiescent (G0) state and self-renewing potential, properties that allow LSCs to evade immune surveillance and resist conventional therapies [11,12]. Alongside its established antiproliferative roles, type I IFNs exert direct cytotoxic and chemosensitizing activities against LSCs [78]. Acute IFN-α exposure in mouse upregulates the transcription and cell-surface expression of stem cell antigen-1 (SCA-1) on HSCs, thereby driving the SCA-1-dependent proliferation and activation of dormant HSCs and immature progenitors within their bone marrow niches [79,80]. Interestingly, chronic type I IFN exposure is known to re-induce HSC quiescence, shielding them from direct and immune-mediated killing [81,82]. Exposure to type II IFN, IFN-γ, impairs HSC self-renewal by inducing the expression of suppressor of cytokine signaling 1 (SOCS1), which subsequently blunts thrombopoietin-mediated STAT5 phosphorylation [83]. Although IFN therapy can directly modulate LSC dynamics, low-dose IFN-γ is linked to the preservation of LSC self-renewal [84,85,86]. In addition, both IFNs drive HSC activation, which risks exhausting dormant HSC reserves and subsequently impairing long-term bone marrow reconstitution and repopulation [79,80]. This double-edged phenomenon warrants further investigation.

IFN therapy not only exhausts the stem cell populations but also promotes blast differentiation toward the mature myeloid lineage, sensitizing them to cell-cycle-targeted chemotherapy and external differentiating agents like DNA methyltransferase (DNMT) inhibitors [87,88,89,90]. IFN-α impedes the secretion of growth-promoting cytokines such as interleukin (IL)-1, IL-6, granulocyte macrophage colony-stimulating factor (GM-CSF) and pro-angiogenic IL-8, and therefore blocks leukemic cell growth and alters the AML microenvironment [91,92,93]. Thus, by interrupting the cell cycle of AML blasts and simultaneously forcing quiescent progenitors into active cycling, these direct anti-leukemic effects may target malignant cells and undermine key resistance mechanisms of AML.

### 2.2. Immunomodulatory Effects

Beyond its direct anti-leukemic effects, IFN therapy has broad and interconnected immunomodulatory effects that collectively shape the immune microenvironment and influence disease progression [94,95,96,97]. Upregulation of major histocompatibility complex (MHC)-I and costimulatory molecules following IFN therapy enhances the immunogenicity of AML cells, leading to more efficient antigen presenting cell (APC) recognition and clearance by cytotoxic T cells (CTLs) and NK cells [98,99]. In addition, IFN-γ-mediated induction of MHC-II expression has also been reported, resulting in an enhanced CD4 T cell response [54,100,101,102].

On dendritic cells (DCs), type I IFN drives phenotypic maturation, promotes migration to lymph nodes and licenses DC for cross-priming of CD8 T cells [99,103]. In the AML setting, DC can even acquire direct cytolytic activity against leukemic targets through TRAIL upregulation, and plasmacytoid and CD8α^+^/CD141^+^ conventional DC subsets serve as both major producers of and responders to type I IFN [47,104,105,106]. In the post-chemotherapy or post-transplant setting, where DC reconstitution may be functionally impaired, these effects on antigen presentation may be especially consequential for the reestablishment of effective immune surveillance [107]. Exposure to IFN-γ has also been shown to critically determine the function of monocyte-derived DCs [108]. On T cells, IFN therapy directly activates CTL-mediated anticancer response independent of CD4+ T helper cells [109]. Type I IFN increases perforin and granzyme B expression, supports memory-CTL survival, sensitizes T cells to IL-2, promotes γδ-T and PR1-specific responses, and even shields antigen-activated CTLs from NK-mediated killing [47,110,111]. Taken together, the capacity of IFN therapy to coordinately restore DC function and enhance cytotoxic T cell and NK cell activity provides a compelling biological rationale for its use as a strategy to strengthen existing therapies and reinstate immune surveillance in AML.

## 3. Clinical Evidence for IFN Therapy in AML

The clinical investigation of IFN therapy in AML spans more than five decades and has focused primarily on IFN-α. Research into IFN-α began with early case reports in the late 1960s and accelerated through the 1980s and 1990s, when multiple clinical studies evaluated its antineoplastic role [46]. The most consequential setback came from two randomized controlled trials. In the first trial, a prospective multicenter Finnish study, patients with *de novo* AML completed six monthly cycles of consolidation therapy before being randomized to maintenance therapy with IFN-α (*n* = 15), monthly five-day courses of thioguanine and cytarabine (*n* = 13), or observation (*n* = 17). Median RFS and median duration of remission were 15 months each for the IFN-α and observation groups, compared to 16 and 18 months for the chemotherapy group. Although median survival was longer in the IFN-α group (33 months vs. 20 months for observation and 26 months for chemotherapy), its five-year survival rate was lower (22% vs. 31% in both comparator arms) [112]. In the UK Medical Research Council (MRC) AML11 trial, 362 of 1314 older patients aged 60 years and older with de novo or secondary AML were randomized to either a 12-month course of low-dose IFN-α-2a maintenance or observation. Among the 182 patients allocated to IFN-α-2a, the rate of death in first complete remission was 5%, matching that of the observation group. Furthermore, five-year outcomes showed no significant benefit for IFN-α-2a over observation, with a relapse risk of 77% vs. 81%, disease-free survival (DFS) of 20% vs. 15%, and OS of 21% vs. 20%, respectively [113]. While a definitive explanation for the observed lack of efficacy remains elusive, this could be in part due to the use of first-generation non-polyethylene glycol-conjugated (pegylated) IFN-α formulations whose dose limitations and short half-life might have rendered sustained receptor engagement pharmacokinetically unachievable, insufficient sample size or treatment period, and the absence of patient stratification [114,115,116,117]. More recent studies have assessed IFNs across multiple AML treatment settings, including induction, maintenance, preemptive/MRD-directed, and salvage therapies, as well as in regimens for special populations unfit for intensive treatment (Table 1).

### 3.1. Case Reports of IFN-α Activity in Frontline Treatment

Berneman et al. reported complete remission (CR) following pegylated IFN-α treatment in a patient with AML transformed from primary myelofibrosis (PMF), demonstrating pegylated IFN-α’s potential effectiveness in AML [50]. The patient was initially on anagrelide and hydroxycarbamide, and both drugs were stopped before reaching CR. Efficacy was demonstrated by normalized white blood cells and absolute neutrophil counts, alongside bone marrow clearance of CD34+ myeloblasts. Furthermore, the antileukemic activity of pegylated IFN-α-2a was corroborated on a molecular level by the eradication of an AML-associated *FLT3* mutation and a significant reduction in *WT1* mRNA levels. At over 10 months of follow-up, the patient sustained a hematological CR. This report describes a case in which pegylated IFN-α offered a well-tolerated alternative to aggressive chemotherapy, achieving clinical improvement without major toxicity.

### 3.2. IFN-α as Maintenance/Prophylaxis Therapy

In one of the earliest investigations of IFN-α in the post-transplant setting, Klingemann et al. conducted a phase 1 dose-escalation study administering recombinant IFN-α to 14 patients at high risk for relapse following transplantation, encompassing a heterogeneous cohort that included AML alongside other hematologic malignancies [118]. High-risk was defined as AML or non-Hodgkin’s lymphoma beyond first CR, ALL at any stage, CML in accelerated or blast phase, or advanced MDS. IFN-α was initiated within 100 days post-transplantation when residual disease burden is presumed to be lowest. The results showed promise, with a two-year RFS of 60% comparing favorably to a historical institutional rate of 21% in diagnostically equivalent patients, providing an early signal that IFN-α administered in the immediate post-transplant period may carry meaningful relapse prevention activity. Nevertheless, myelosuppression remained a significant concern, with dose-limiting neutropenia and thrombocytopenia occurring in the majority of patients at higher dose levels. In terms of immunological safety, three patients developed non-dose-dependent, steroid-sensitive acute GvHD after receiving IFN-α, which led to treatment discontinuation. These safety signals underscore the challenge of safely administering IFN-α in an already hematopoietically vulnerable post-transplant population.

Later on, Magenau et al. also explored pegylated IFN-α as a prophylactic treatment to reduce post-transplant relapse risk among patients with AML at high risk of relapse, defined as active AML at HSCT or morphologic remission with very poor-risk cytogenetic/molecular features, or primary induction failure [51]. In this phase 1/2 trial (NCT02328755) of 36 patients treated with pegylated IFN-α, the cumulative incidence of relapse (CIR) was 39% at six months and remained stable through one year post-transplant. Of note, pegylated IFN-α maintained a manageable safety profile without substantially increasing transplant-related toxicity, demonstrating a six-month non-relapse mortality rate of 13% and a low 11% incidence of grade 3–4 acute graft versus host disease (GvHD). The authors reported two-year OS and DFS of 33% and 28%, respectively, comparing favorably to a contemporaneous non-enrolled cohort from the same institution that had a two-year OS of only 15%. The authors concluded that prophylactic pegylated IFN-α is feasible with a manageable safety profile.

The efficacy of preemptive IFN-α therapy in patients with favorable-risk AML who achieve CR but remain MRD-positive following consolidation was investigated by Jiang and colleagues, an approach that is now being validated in an ongoing Phase 3 randomized clinical trial with an observational control arm (NCT06802718). In this retrospective cohort of 84 patients, the 42 patients who received IFN-α-2b maintenance therapy (3 million IU thrice weekly for 12–18 months) were associated with longer RFS and OS. At four years, the estimated RFS was 86.8% in the IFN-α group compared to 55.7% in the control group (*p* = 0.007). The four-year estimated OS was higher with IFN-α (94.4% vs. 76.4%; *p* = 0.04). Furthermore, multivariable analysis showed that IFN-α therapy remained associated with longer RFS (*p* = 0.004). These findings suggest a potential role for IFN-α as an effective MRD-directed preemptive therapy to mitigate relapse risk in favorable-risk AML following consolidation that warrants prospective confirmation [115].

### 3.3. IFN-α as MRD-Directed Preemptive Therapy

MRD-directed preemptive therapy represents the largest contemporary clinical evidence base for IFN-α use in AML.

In a prospective study, Mo et al. evaluated preemptive IFN-α as a viable alternative to DLI for managing MRD following allo-HSCT in patients with acute leukemia and myelodysplastic syndrome [119]. Treatment with subcutaneous IFN-α achieved a 90% MRD clearance rate, with outcomes comparable to standard DLI interventions. Notably, one-year outcomes for relapse (27.3%), non-relapse mortality (4.5%), and DFS (68.2%) in the IFN-α cohort matched those of the DLI group, and the therapy notably avoided the severe hematologic toxicities often associated with DLI. However, the potent immunostimulatory graft-versus-leukemia (GvL) effect induced by IFN-α was accompanied by a significantly higher and more rapid onset of chronic GvHD, affecting 90.9% of recipients compared to 62.9% in the DLI cohort. Two registry studies (NCT02027064 and NCT02185261) also demonstrated the durable efficacy of this preemptive approach [120]. Among post-transplant patients who received preemptive IFN-α for MRD positivity, the six-year OS and DFS reached 88.3% and 83.1%, respectively. Importantly, this intervention translated to a six-year CIR rate of 13.0% and a remarkably low non-relapse mortality (NRM) rate of 3.9%. Furthermore, while chronic GvHD was frequently observed, the incidence of severe chronic GvHD was only 10.4%, suggesting that preemptive IFN-α may achieve durable MRD response with a manageable long-term safety profile. These results suggest that IFN-α therapy may help mitigate the adverse prognostic impact associated with post-transplant MRD positivity in AML, with survival outcomes approaching levels reported in MRD-negative patients.

In a retrospective study of 151 pediatric post-transplant AML patients, the authors demonstrated that preemptive IFN-α, directed by sequential *WT1* monitoring, achieved MRD clearance in 64% of treated patients. Importantly, patients who converted to MRD negativity after IFN-α achieved a five-year CIR of 8.3% and DFS of 91.7%, which were comparable to the 11.3% and 80.8% observed in those who were MRD-negative from the outset. Both cohorts had significantly superior outcomes compared to untreated MRD-positive patients, who experienced a CIR of 36.2% and a DFS of 59.9% [121].

Patients with t(8;21) AML represent a subgroup in which MRD monitoring is standardized via the quantification of *RUNX1-RUNX1T1* transcript levels. In a prospective registry study (NCT02027064) evaluating 42 patients with t(8;21) AML who developed MRD post-transplant, preemptive IFN-α treatment demonstrated significant efficacy [122]. The one-year OS and DFS were 92.5% and 92.4%, respectively. Furthermore, the one-year CIR was only 7.2%, supporting IFN-α activityi against MRD within this specific genetic context. A subsequent study by the same group evaluated the efficacy of IFN-α stratified by the magnitude of MRD burden at the time of detection [123]. The authors reported that MRD clearance rates were highest among patients with low-level *RUNX1-RUNX1T1* transcript positivity (87.5%), decreasing progressively in those with intermediate (58.1%) and high-level (22.2%) positivity. These findings underscore the critical importance of intervention timing, indicating that early preemptive therapy yields superior results. Meanwhile, among patients with low- to intermediate-level MRD, the two-year OS rate was markedly higher in the IFN-α cohort (87.6%) compared to those receiving DLI (55.6%). However, this survival advantage was not observed in patients with high-level MRD, where clinical outcomes between the IFN-α and DLI groups were comparable.

Real-world analyses reported findings consistent with these registry findings. In a post-transplant AML cohort, Fan et al. reported that 78.2% of patients achieved MRD negativity at two years following preemptive IFN-α [124]. This cohort demonstrated three-year OS (84.2%) and DFS (76.9%), alongside notably low incidences of relapse (20.9%) and severe chronic GvHD (6.2%). Similarly, a case–control study by Lin et al. in a broader cohort of acute leukemia patients demonstrated that treatment response without progression to hematological relapse was achieved by 80.6% of patients in the preemptive IFN-α group, compared to only 32.8% in the non-IFN-α group [125]. Consequently, the two-year CIR was 31.6% and 61.2% in the preemptive IFN-α and non-IFN-α groups, respectively, translating to roughly doubled rates of DFS (57.4% vs. 28.4%) and OS (67.6% vs. 32.9%). Interestingly, treatment efficacy strongly correlated with the induction of GvHD. among IFN-α-treated patients, 58.1% developed GvHD, which was associated with a significantly higher response rate (88.9%) compared to those who did not (53.8%), indicating that the immunomodulatory effect of IFN-α might cause GvL and GvHD simultaneously.

Beyond monotherapy, a phase 2 study of 47 AML/MDS patients with post-transplant MRD positivity found that pre-emptive azacitidine plus IFN-α achieved response rates of 72.3% after cycle one, rising to 77.2% by cycle five, with three-year OS, DFS, and EFS of 69.5%, 60.4%, and 35.7%, respectively. Fewer than six treatment cycles and absence of chronic GvHD were independently associated with worse outcomes, while toxicity remained low, with no severe myelosuppression observed [126]. These findings suggest that azacitidine plus IFN-α may offer a feasible and tolerable pre-emptive strategy to reduce relapse risk in MRD-positive patients following allo-HSCT.

Although the efficacy and safety profile of preemptive IFN-α is supported by several clinical studies, most of this evidence originates from a single institution utilizing haploidentical transplant protocols. Variations in other transplant-related factors, including conditioning regimen, graft sources and GvHD prophylaxis, might also influence clinical outcomes. Nevertheless, this clinical research constitutes a mature and internally consistent dataset available for preemptive IFN-α in post-transplant AML.

### 3.4. IFN-α as Salvage Therapy in Relapsed/Refractory AML

Early clinical experience with salvage IFN-α was largely derived from individual case reports and its integration into broader combination strategies. Singhal et al. described a patient with secondary AML relapsing after allo-HSCT who was treated with both interleukin-2 (IL-2) and IFN-α, although IL-2 was discontinued early due to poor tolerability. IFN-α alone was able to reduce marrow blasts (fluctuating between 1 and 6% compared to 10% in bone marrow after transplant) and maintain remission with stable hematological parameters. Following IFN-α discontinuation due to the development of limited chronic GvHD, disease remission was sustained for at least 26 months with methotrexate and other treatments [127]. A later case report by Gesundheit et al. further supported the potential role of IFN-α in post-transplant AML relapse, demonstrating durable complete remission following combined DLI and IFN-α administration, with the authors attributing IFN-α’s contribution to immunomodulatory enhancement of the GvL response [128].

Building upon early case reports regarding IFN-α use in the salvage setting, further investigations have expanded to retrospective studies and clinical trials. Mo et al. investigated IFN-α as salvage therapy in 24 patients with AML or other hematological malignancies who had persistent MRD after MRD-directed DLI following allo-HSCT [129]. The study demonstrated that compared to patients with persistent MRD who did not receive salvage IFN-α, the IFN-α-treated group had significantly lower two-year CIR (35.9% vs. 64.7%, *p* = 0.007) and superior DFS (54.3% vs. 29.4%, *p* = 0.004), with a trend toward improved OS (68.0% vs. 41.7%, *p* = 0.076). In addition, clinical outcomes in the IFN-α salvage group were comparable to those who received salvage DLI, suggesting IFN-α as a viable alternative when repeat DLI is not feasible. The two-year NRM was low at 8.3%, with GvHD being the most common reason for treatment discontinuation. Multivariate analysis confirmed that salvage IFN-α treatment independently reduced relapse risk and improved DFS, supporting its role as an effective salvage strategy for MRD clearance after DLI failure. A prospective phase 1/2 trial (ACTRN12612000728831) evaluating pegylated IFN-α, conducted by Henden et al., further explored the use of IFN-α in salvage setting [49]. In 29 patients relapsing after allo-HSCT across a range of hematological malignancies, with AML being the most common diagnosis (45%). Following reinduction chemotherapy, pegylated IFNα was administered with optional DLI in the absence of GvHD at 6 weeks. The two-year OS and PFS were 31% and 24%, respectively. Chronic GvHD developed in 62% of patients, and these individuals exhibited a significantly better PFS compared to those who did not (median 284 vs. 43 days). Treatment of post-reinduction MRD yielded clinical responses in 50% of cases, leading to deep remissions with complete MRD eradication in half of the responding cohort. Collectively, both studies provide encouraging evidence supporting the role of IFN-α in the post-transplant AML setting.

### 3.5. Other IFNs as Salvage Therapy

Although clinical evidence for the application of IFN-γ in AML remains relatively sparse, a phase 1 first-in-human trial (NCT04628338) of IFN-γ therapy with or without DLI in patients with relapse after allo-HSCT showed that four of six patients with relapsed AML or myelodysplastic syndrome who received IFN-γ plus DLI achieved MRD-negative complete remission and full donor hematopoietic recovery, and further investigation in a phase 2 trial (NCT06529731) extending to a broader allo-HSCT population is underway [130]. Conversely, emapalumab, a monoclonal antibody that neutralizes IFN-γ, is being evaluated in a phase 1 trial (NCT06996119) in combination with post-transplant cyclophosphamide, tacrolimus, and mycophenolate mofetil to prevent GvHD in patients with MDS and AML, underscoring the complex and context-dependent role of IFN-γ and the need for careful investigation.

On the other hand, a novel oncolytic virotherapy based on vesicular stomatitis virus engineered to express IFN-β and the sodium-iodide symporter is undergoing clinical evaluation [131]. The IFN-β transgene is designed to exert IFN-mediated effects that protect nonmalignant tissues while also promoting T-cell cross-priming during viral infection [132]. This platform has demonstrated a favorable safety profile in canine studies [133]. The ongoing phase 1 trial (NCT03017820) is assessing its safety and optimal dosing in patients with relapsed or refractory hematologic malignancies, including AML. This study may also provide further insight into the therapeutic role of IFN-β in modulating antitumor immunity and protecting nonmalignant tissues during oncolytic virotherapy.

Although signals of clinical activity have been documented throughout the AML treatment journey, the overall accumulated evidence remains inconsistent. As a result, its use continues to be controversial and IFN-α never achieved integration into standard AML practice. In parallel, clinical evaluation of IFN-β and IFN-γ remains sparse. The therapeutic utility of IFN-γ continues to be debated given its pleiotropic and context-dependent functions, with both immunostimulatory and inhibitory strategies currently under investigation.

**Table 1:** Clinical findings of IFN therapy in AML patients.

Study	Study Type	Population	N	IFN Formulation/Regimen	Comparator	Primary Findings	Major Toxicities
Frontline Therapy							
Berneman et al. 2010 [50]	Case report	AML transformed from primary myelofibrosis	1	Pegylated IFN-α-2a	NA	Continued CR > 10 m	Mild transient arthralgias
Maintenance/Prophylactic Therapy							
Klingemann et al. 1991 [118]	Phase 1 study	High-risk hematologic malignancies (AML, NHL, ALL, CML, MDS)	14	IFN-α-2b	NA	2 yr RFS 60%; MTD 1.0 × 10^6^ IU/m^2^/day	Dose-limiting myelosuppression at ≥1.5 × 10^6^ IU/m^2^. aGVHD in 3/11 allograft recipients
Magenau et al. 2021 [51]	Phase 1/2 study	High-risk AML (refractory or poor-risk cytogenetics)	36	Pegylated IFN-α-2a	NA	6 m relapse 39%; 2 yr OS 33%; DFS 28%	Grade ≥ 3 SAEs 25%; MTD 180 µg; grade 3–4 aGVHD 11%; NRM 13%
Jiang et al. 2021 [115]	Retrospective	Favorable-risk AML	84	IFN-α-2b	Without IFN	4 yr estimated RFS 86.8% vs. 55.7%; estimated OS 94.4% vs. 76.4%; IFN-α as independent predictor of RFS (*p* = 0.004)	Transient fever 69.1%; grade ≥ 3 thrombocytopenia 11.9%
MRD-Directed Preemptive Therapy							
Mo et al. 2015 [119]	Prospective study	Acute leukemia in first or second CR without t(9;22) or MDS-refractory anemia with excess blasts underwent transplant	67	IFN-α-2b	DLI	90% MRD clearance rate; 1 yr relapse 27.3%; DFS 68.2%; outcomes comparable to DLI	NRM 4.5%; cGvHD 90.9% vs. 62.9%
Deng et al. 2021 [121]	Retrospective study	Post-transplant pediatric AML	151	IFN-α-2b	NA	MRD negativity 64%; among converters 5 yr CIR 8.3% vs. 36.2%; DFS 91.7% vs. 80.8%, comparable to MRD-negative patients and better than MRD+ patients received no intervention CIR 36.2%; DFS 59.9%	Persistent MRD in 9/25 (3 relapsed); GvHD higher with IFN vs. DLI; NRM comparable
Mo et al. 2018 & Fan et al. 2022 [122,123]	Prospective & retrospective study	Post-transplant MRD+ t(8;21) AML	42	IFN-α-2b	DLI	1 yr OS 92.5%; DFS 92.4%; CIR 7.2%; among low- to intermediate- MRD, 2 yr OS rate 87.6% vs. 55.6%	Transient fevers in all patients; 2 grade ≥ 3 hematologic toxicities; 4 grade ≥ 3 infectious toxicities
Fan et al. 2023 [124]	Retrospective study	Post-transplant MRD+ AML	247	IFN-α-2b	NA	2 yr MRD negativity 78.2%; 3 yr relapse 20.9%; DFS 76.9%; OS 84.2%	Total/severe cGVHD 53.2%/6.2%. Severe cGVHD independently associated with NRM (HR 9.98). Infection in 27.9% (10 infection-related deaths)
Lin et al. 2018 [125]	Retrospective study	Post-transplant acute leukemia	98	IFN-α-2b	Without IFN	Response rate 80.6% vs. 32.8%; 2 yr CIR 31.6% vs. 61.2%; 2 yr DFS 57.4% vs. 28.4%; 2 yr OS 67.6% vs. 32.9%	Mild fever, myalgia 41.9%; anorexia 25.8%; 5 grade 1–2 cytopenias; 6 aGVHD (2 grade 2, resolved); 12 limited cGVHD
Huang et al. 2024 [126]	Phase 2 study	Post-transplant MRD+ AML/MDS	47	IFN-α or pegylated IFN-α-2b + Azacitidine	NA	3 yr OS 69.5%; DFS 60.4%; EFS 35.7%; CIR 36.5%;	NRM 4.3%; reversible grade 3–4 neutropenia 27.7%; thrombocytopenia 23.4%; CMV reactivation 6.4%; EBV reactivation 23.4%
Salvage Therapy							
Singhal et al. 1997 [127]	Case report	Post-transplant secondary AML relapse	1	IFN-α-2b + IL-2 (temporarily)	NA	Marrow blasts reduction from 10% to 1–6%; hematologic remission	large granular lymphocytosis; eosinophilia; elevated serum ALP
Gesundheit et al. 2009 [128]	Case report	t(8;21) AML	1	IFN-α-2a + DLI	NA	Stable hematologic parameters; physically well > 24 m	Steroid-sensitive GvHD
Mo et al. 2019 [129]	Retrospective study	Post-transplant AML or other hematological malignancies with persistent MRD after MRD-directed DLI	24	IFN-α-2b	Without IFN	2 yr CIR 35.9% vs. 64.7%; DFS 54.3% vs. 29.4%; OS 68.0% vs. 41.7%	2 yr NRM 8.3%; grade ≥ 3 aGvHD 8.3%
Henden et al. 2019 [49]	Phase I/II study	Post-transplant AML, MDS, ALL relapse	29	Pegylated IFN-α-2a	NA	2 yr OS 31% (median 330 days); 2 yr PFS 24% (median 184 days); GvHD associated with improved PFS (*p* = 0.024)	Grade ≥ 3 AEs 38%; 2 fatal (sepsis, GVHD); cytopenias most common dose-limiting toxicity
Ito et al. 2025 [130]	Phase I study	AML, MDS	7	IFN-γ monotherapy, followed by DLI + concurrent IFN-γ	NA	4 MRD-CR; median OS of 579 days in responders	Steroid-sensitive grade 1–2 GvHD, ICANS, IPS

AE, adverse event; aGVHD, acute graft-versus-host disease; ALL, acute lymphoblastic leukemia; ALP, alkaline phosphatase; AML, acute myeloid leukemia; cGVHD, chronic graft-versus-host disease; CIR, cumulative incidence of relapse; CML, chronic myeloid leukemia; CMV, cytomegalovirus; CR, complete remission; DFS, disease-free survival; DLI, donor lymphocyte infusion; EBV, Epstein–Barr virus; EFS, event-free survival; GVHD, graft-versus-host disease; HR, hazard ratio; ICANS, immune effector cell-associated neurotoxicity syndrome; IFN, interferon; IL-2, interleukin-2; IPS, idiopathic pneumonia syndrome; IU, international units; MDS, myelodysplastic syndrome; MRD, measurable (minimal) residual disease; MTD, maximum tolerated dose; N, number of patients; NA, not applicable; NHL, non-Hodgkin lymphoma; NRM, non-relapse mortality; OS, overall survival; PFS, progression-free survival; RFS, relapse-free survival; RR, response rate; SAE, serious adverse event.

## 4. Safety and Toxicity

The safety profile of IFN therapy is frequently characterized by a “double-edged sword” effect, where the immunomodulatory effects required to drive the GvL response concurrently elevates the risk of GvHD. Chronic GvHD is the primary safety concern, with studies reporting that cumulative incidences can exceed 60% [119]. However, this development often closely correlates with clinical response and reduced relapse, underscoring the mechanistic overlap between GvL and GvHD [49,124,125]. A study by Mo et al. further showed that only mild or moderate GvHD, rather than severe GvHD, is associated with reduced relapse risk and improved DFS [134]. This highlights the clinical necessity of careful balancing the GvL effect against GvHD and determining the optimal timing and duration for therapeutic intervention.

Beyond immune complications, standard short-acting IFNs can induce systemic and hematologic adverse events [117]. To mitigate the risk and optimize the therapeutic window, recent advancements have focused on long-acting formulations, particularly pegylated IFN-α. Safety differences could be rooted in the structural design and its subsequent impact on pharmacokinetic profiles. Traditional recombinant IFNs are small proteins subject to rapid systemic clearance, necessitating frequent dosing that leads to volatile peak-and-trough serum levels, which may drive myelosuppression and bothersome constitutional symptoms. In contrast, pegylated IFNs employ PEG conjugation to increase the molecule’s hydrodynamic size and thereby extends the cytokine’s half-life, yielding stable, continuous pharmacokinetics that allow for less frequent dosing [135]. This steady-state exposure is expected to attenuate peak-dose constitutional and hematologic toxicities while sustaining the continuous immunological pressure required for robust GvL effects [49,51]. Newer pegylated IFN-based therapy such as ropeginterferon alfa-2b, utilized mono-pegylation, offering a more homogeneous single isomer structure that may minimize toxicities via less frequent dosing in healthy volumteers [135]. By offering enhanced tolerability and a safer profile for patients, advanced long-acting agents represent a potential strategy in AML therapy and an emerging avenue for future clinical studies.

## 5. Conclusions

The repositioning of IFN therapy in AML represents a critical intersection of historical pharmacology and modern cancer immunology. This review synthesizes the evolving landscape of IFN therapy in AML, bridging its profound biological rationale with stratified clinical applications. IFNs exert direct antileukemic activity by targeting AML blasts and LSCs, while simultaneously leveraging its immunomodulatory properties to remodel the functional status of immune cells within the leukemia microenvironment. These therapeutic mechanisms can be further modulated by the temporal dynamics of IFN exposure, highlighting the importance of strategic scheduling. While acute IFN therapy can be exploited to facilitate direct HSC killing, chronic exposure appears to exert a protective effect by restoring HSC quiescence, thereby shielding these cells from cytotoxic attack [80,81,82,136]. By evaluating the role of IFN therapy across the treatment continuum, this work provides a framework for prioritizing future clinical investigation. Aggregated evidence underscores the potential clinical benefits of both IFN monotherapy and combination regimens across multiple clinical contexts, especially in AML preemptive and maintenance settings. In these scenarios, patient cohorts with low-level molecular MRD, those with core-binding factor AML, particularly *RUNX1::RUNX1T1*, and those with adequate post-transplant immune reconstitution, may represent favorable candidates for IFN therapy. Biologically, preserved IFNAR-JAK-STAT signaling, retained HLA/MHC expression and proper T- and NK-cell composition may serve as permissive conditions, although their utility as predictive biomarkers has yet to be clinically validated. IFN is a mechanistically compelling combination partner capable of directly targeting LSCs and priming the immune microenvironment alongside contemporary treatment agents. Given the acute nature of AML, further clinical evidence is necessary to identify eligible patient cohorts and validate the efficacy of the combination strategies. Nevertheless, standardizing inconsistent IFN dosing regimens, establishing universal patient selection criteria, and identifying biomarkers of response and resistance remain critical challenges to balancing the IFN’s dual effects and optimizing clinical outcomes. The interpretations drawn in this review must also be contextualized within the inherent limitations of the existing literature. Much of the available clinical evidence is derived from individual case reports, nonrandomized studies, and early-phase trials with heterogeneity across these studies regarding baseline patient characteristics, risk profiles, transplant conditioning regimens, donor types, and treatment protocols. Notably, most of the clinical evidence summarized in this review supporting IFN therapy as an MRD-directed preemptive therapy originates from a single research group utilizing haploidentical transplant protocols. This variability complicates cross-study comparisons and limits definitive meta-analyses. Additionally, a recent study also indicated that endogenous IFN signaling regulates leukemic dormancy, affecting sensitivity to cell-cycle-active agents [137]. Further translational research and well-designed multi-center studies with standardized criteria are warranted to strengthen the evidence base and inform the development of clinical guidelines.

## Data Availability

No new data were created or analyzed in this study. Data sharing is not applicable to this article.
